# Partner notification

**DOI:** 10.1016/j.mpmed.2014.03.013

**Published:** 2014-06

**Authors:** Helen Ward, Gill Bell

**Affiliations:** **Helen Ward PhD FFPH FRCP MBChB MSc** is a Professor of Public Health, Imperial College London, UK. Competing interests: none declared; **Gill Bell MA BA (Hons)** is a Nurse Consultant Sexual Health Adviser at Sexual Health Sheffield, Sheffield Teaching Hospitals NHS Foundation Trust, Sheffield, UK. Competing interests: none declared

**Keywords:** Contact tracing, HIV prevention and control, HIV transmission, sexual partners, sexually transmitted diseases

## Abstract

Partner notification is an essential part of case management for sexually transmitted infections. Done correctly it reduces persistent or recurrent infection in the index patient, identifies previously undiagnosed infections, and may thus contribute to reduced transmission in the population. The effectiveness of patient referral of partners can be enhanced through the provision of written information and easy access to tests and medication. A recent systematic review of partner notification found that enhanced partner therapy (helping get treatment to partners more rapidly) reduced re-infection in the index case by almost 30% compared with simple patient referral. Provider referral, where the healthcare worker contacts partners directly, can also be effective, and provides an important service for patients who are wary of informing partners themselves. Partner notification services should be available for all patients found to have a sexually transmitted infection, whether the diagnosis is made in specialist settings, or in primary or community-based care. For patients with HIV, partner notification should be addressed when the infection is first diagnosed and revisited for subsequent partners. Access to specialist partner notification services is an important part of any sexual healthcare system. The professional competencies required to undertake partner notification have now been clearly defined.

**What's new?**

• Enhanced partner therapy: providing easy access to tests and treatment for partners without requiring them to attend clinic for a consultation appears to improve outcomes; in some places (outside the UK) patients can deliver treatment to partners, in others this has to be facilitated by healthcare workers including pharmacists
• Electronic communication: text messaging and web-based systems can be used to facilitate partner notification

## Overview

Partner management is a key part of patient care and infectious disease control for sexually transmitted infections (STIs). It includes notification of the partner and provision of appropriate tests and treatment. Partner notification (or contact tracing) is the process by which the sexual contacts of a patient with an STI are informed that they may be at risk. They are then offered screening, and treatment if indicated. The aim is to find and treat undiagnosed, often asymptomatic, infection. This helps to reduce re-infection in the index patient, and the spread of STIs in the community. Partner notification is a method of targeted case-finding that leads to early diagnosis and treatment, reduces transmission, prevents sequelae and provides an opportunity to discuss safer sex. The term ‘partner notification’ has generally replaced ‘contact tracing’ because it better reflects the two basic approaches – namely, patients informing their partners themselves, and health workers contacting partners directly.

## Approaches

Ideally, patients should be offered a choice of approaches to partner notification (Table 1).

**Table 1:** Definitions

Patient referral	The index case is responsible for notifying their sexual partner(s) and referring them to a clinic.
Simple patient referral	A health professional advises the index case that their sexual partners need to be treated and explains how to do it.
Enhanced patient referral (EPR)	Simple patient referral plus one or more of: written information about the infection to be given to partners, use of a website, sampling kits for index cases to give to partners.
Accelerated partner therapy (APT)	Sexual partners are offered access to antibiotic treatment through a telephone consultation with a clinician or pharmacist to assess eligibility for treatment, without requiring a face-to-face consultation. This is a modification of expedited partner therapy (see below) that complies with UK prescribing regulations.
Expedited partner therapy (EPT)	Index patients are provided with antibiotics or a prescription to give to their sexual partners without the need for a consultation with a health professional. Developed in the USA, not legal in UK.
Provider referral	A health adviser notifies the partner of their possible exposure without identifying the index patient.
Contract referral	The index case agrees to notify partners within a specified time period, and if this is not done the health adviser will proceed to provider referral.

Table adapted from Ref. ^7^.

### Patient referral

In this option, the index case informs their own sexual partners of the need for tests and/or treatment. This is the preferred approach for the majority of patients^1^ and may be the only option in non-specialist settings.^2^ Trials have shown that patient referral may be more effective if patients take written information,^3^ home-sampling kits,^4^ or even medication for the partner without a consultation (expedited partner therapy, EPT).^5^ In the UK, where medication cannot be prescribed without a consultation, an alternative method, accelerated partner therapy (APT), has been developed that allows partners to collect treatment and testing kits from a clinic or pharmacy following a telephone or face-to-face consultation with the prescriber.^6^

A recent systematic review of partner notification found that enhanced partner therapy using APT or EPT reduced re-infection in the index case by almost 30%.^7^ However, they were not significantly better than enhanced partner referral (EPR) in which specific support is offered to patients in bringing their partners into contact with health services. Web-based partner notification services are also available for men who have sex with men to contact partners anonymously via dating sites, text message or email.^8^ A follow-up telephone phone call by prior agreement also improves outcomes,^9^ providing an opportunity to repeat the offer of provider referral if difficulties have been experienced.

### Provider referral

In this option, a healthcare worker informs the partner confidentially, without disclosing the patient's identity. The infection is named only if the patient gave permission. Provider referral is more often requested for casual or former partners whom the patient prefers not to approach. The service protects patients from potential harms, such as embarrassment, a damaged reputation or abuse. From a public health perspective, provider referral contributes to control by reaching partners who may not otherwise be informed. Recent studies have found that provider referral is more frequently requested for casual partners,^7^ who play a more significant role in onward transmission: the number of partners of who need to be treated to interrupt transmission is 1.1 for casual partners, compared with 2.5 for regular partners.^10^ Provider referral is usually undertaken by a specialist sexual health adviser based in a sexual health clinic, and recent guidelines recommend that this approach is made available wherever STIs are diagnosed through the development of a community-based partner notification service.^11^

### Contract or conditional referral

In this option, the patient has an agreed period of time to refer the partner for care before the healthcare worker makes contact directly. This may be favoured by patients who are unsure whether they can inform partners themselves.

## Indications

Partner notification should be undertaken for all those with a treatable STI (including gonorrhoea, chlamydia, syphilis, trichomoniasis, chancroid, hepatitis B, HIV) including non-genital sites (e.g. ocular chlamydia). In some viral infections (e.g. genital herpes, genital warts), the benefit of notification to partners is less clear because of difficulties in diagnosis and treatment, but it is still useful to warn them of possible exposure.

## Who?

The importance of notifying partners should be discussed as soon as possible – preferably when the diagnosis is disclosed. Patients can be offered support and assistance from a specialist sexual health adviser or, where this is not possible, partner notification needs to be addressed by the clinician explaining the diagnosis. Specialist support with partner notification should always be arranged for patients with serious infections such as HIV or syphilis. Healthcare workers providing partner notification should have documented competencies appropriate to the care given. These competencies should correspond to the content and methods described in the Society of Sexual Health Advisers (SSHA) National Sexual Health Advisers Competencies – Competency Record Book.^12^, ^13^

## How?

**Figure 1:**
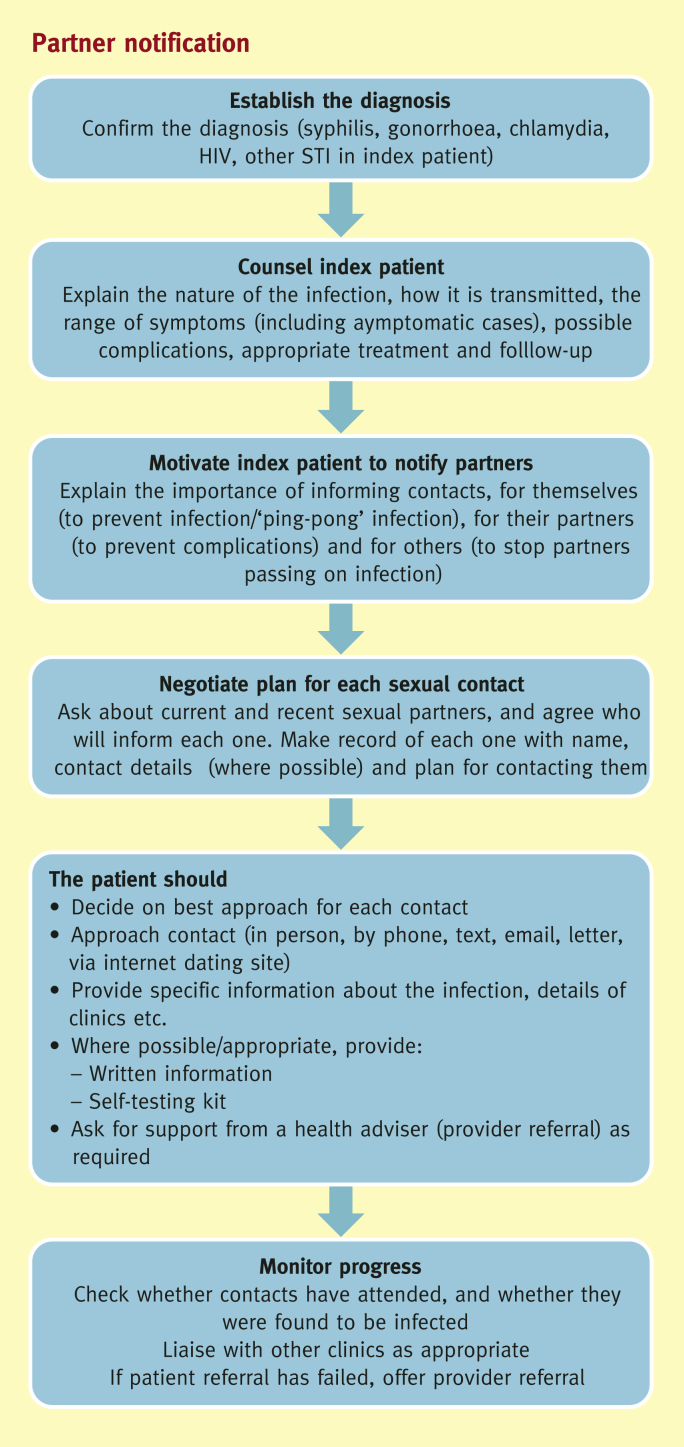
Partner notification.

The process of partner notification is shown in Figure 1. It is theoretically straightforward, but requires a high degree of sensitivity and understanding. Patients are concerned about confidentiality, the impact on their relationship, and how to broach the subject. The possible negative impact of disclosing an STI to a partner should be considered, including the risk of stigma or abuse following disclosure. Taking a detailed sexual history is a skilled process, requiring a non-judgmental and relaxed approach. Too often, sex is discussed in hushed tones with a hint of embarrassment that does little to put the patient at ease. It is easiest to start with the most recent partner, and then work back to earlier contacts. When there is any doubt about the source of the infection, it is usual to ask about all partners within the last 3 months for gonorrhoea and 6 months for chlamydia. For syphilis and HIV the look-back period varies depending on history and stage of infection. Details of look-back periods are covered in the British Association for Sexual Health and HIV (BASHH) Statement on Partner Notification.^12^

## Rights and responsibilities

The clinician must strike a balance between responsibility to the individual patient and the protection of others. In most cases, the balance lies with the patient, and ensuring that he or she is treated, returns for a test of cure, and is not deterred from sexual health services in the future by a heavy-handed approach to partner notification. Co-operation with partner notification is voluntary in the UK: patients are not required by law to inform partners as they are in some jurisdictions, such as Sweden. However, partners who are put at continuing risk of exposure to a serious STI such as HIV may be informed of their risk, without the patient's consent if necessary, to protect them from becoming infected. The decision to breach patient confidentiality must be taken in line with professional guidance on disclosure.^14^

## Does it work?

Enhanced partner notification interventions including APT, EPT and EPR with written information and sampling kits for partners are more effective than simple patient referral in terms of the numbers of partners informed and/or treated, and in reducing persistent or recurrent infection in the index patient.^7^, ^15^ Partner notification is likely to reach only a proportion of contacts, often missing those who sustain transmission in the population, as it is more successful in longer-term relationships and less so in short-term casual contacts.^16^ As a result, the contribution of partner notification to control of transmission in the population is limited, although it can be particularly effective in controlling outbreaks and targeting screening and management towards those most likely to be infected. Mathematical models suggest that improving partner notification could be highly cost-effective in terms of cost per infection diagnosed when compared with expanding coverage of screening, for example.^17^ In specialist units such as sexual health or genitourinary medicine clinics the ‘outcomes’ from partner notification are regularly audited against national professional standards.^12^

## The future

Innovative uses for communications technologies, including web-based notification systems, have the potential to reach partners who are increasingly being met through the internet. New diagnostic methods mean that self-taken samples and even self-tests are likely to be another tool in the management of partners. As with all use of these new technologies, it is important to remember that contact with healthcare workers remains important for many people in order to initiate notification of additional partners, to detect co-morbidities and to provide expert advice and support.

**Practice points**

• Primary care and community settings: partner notification should be supported in all settings where STI and HIV are diagnosed, which could be through the development of a local partner notification bureau that supports services in primary care, community services and chlamydia screening programmes
• Written information about the specific infection should be provided for the patient to give to their partner(s)
• Provider referral should be offered to patients where appropriate
• Partner notification should be supported by healthcare workers with the appropriate competencies, which have now been clearly defined
